# Central Nervous System Stimulants Limit Caffeine Transport at the Blood–Cerebrospinal Fluid Barrier

**DOI:** 10.3390/ijms23031862

**Published:** 2022-02-07

**Authors:** Kei Ikeda-Murakami, Naoto Tani, Tomoya Ikeda, Yayoi Aoki, Takaki Ishikawa

**Affiliations:** 1 Department of Legal Medicine, Osaka City University Medical School, 1-4-3 Asahi-machi, Abeno, Osaka 545-8585, Japan; tani.naoto@med.osaka-cu.ac.jp (N.T.); ikeda.tomoya@med.osaka-cu.ac.jp (T.I.); aoki.yayoi@med.osaka-cu.ac.jp (Y.A.); takaki@med.osaka-cu.ac.jp (T.I.); 2 Forensic Autopsy Section, Medico-Legal Consultation and Postmortem Investigation Support Center, c/o Department of Legal Medicine, Osaka City University Medical School, 1-4-3 Asahi-machi, Abeno, Osaka 545-8585, Japan

**Keywords:** caffeine, stimulants, blood–cerebrospinal fluid barrier (BCSFB), BCSFB model, choroid plexus, vacuolation, GC/MS

## Abstract

Caffeine, a common ingredient in energy drinks, crosses the blood–brain barrier easily, but the kinetics of caffeine across the blood–cerebrospinal fluid barrier (BCSFB) has not been investigated. Therefore, 127 autopsy cases (Group A, 30 patients, stimulant-detected group; and Group B, 97 patients, no stimulant detected group) were examined. In addition, a BCSFB model was constructed using human vascular endothelial cells and human choroid plexus epithelial cells separated by a filter, and the kinetics of caffeine in the BCSFB and the effects of 4-aminopyridine (4-AP), a neuroexcitatory agent, were studied. Caffeine concentrations in right heart blood (Rs) and cerebrospinal fluid (CSF) were compared in the autopsy cases: caffeine concentrations were higher in Rs than CSF in Group A compared to Group B. In the BCSFB model, caffeine and 4-AP were added to the upper layer, and the concentration in the lower layer of choroid plexus epithelial cells was measured. The CSF caffeine concentration was suppressed, depending on the 4-AP concentration. Histomorphological examination suggested that choroid plexus epithelial cells were involved in inhibiting the efflux of caffeine to the CSF. Thus, the simultaneous presence of stimulants and caffeine inhibits caffeine transfer across the BCSFB.

## 1. Introduction

Recently, the increasing consumption of energy drinks in adolescents and young adults has been regarded as a problem [1]. Furthermore, poisoning-related deaths caused by high doses of caffeine, a major component of energy drinks, have been reported [2,3,4]. Energy drinks contain a large amount of caffeine, and the increasing consumption of energy drinks by young persons is a problem. Drug use in young persons along with the consumption of energy drinks is a problem. Moreover, the link between energy drinks and drug use that affects the central nervous system (CNS) has been widely discussed for some time [5,6,7,8]. The consumption of energy drinks is positively correlated with the use of drugs, such as amphetamines [5,8], and recent studies have found that energy drinks consumers use a high rate of CNS stimulants [6]. Furthermore, studies of people with a history of drug use have reported that 78.6% of the subjects deliberately ingested caffeine in the past. As a means of intake, 69.2% consumed energy drinks, 24.5% took tablets containing caffeine as the main ingredient, and 4.9% used intranasal spray [8]. Although there is a certain link between energy drinks and drug use, whether each affects the CNS differently depending on the intake concentration when using a CNS stimulant alongside caffeine is unclear.

The relationship between stimulants and caffeine has been investigated in the brain [9,10,11], but not in the transport system. The blood–brain barrier (BBB) and the blood–cerebrospinal fluid barrier (BCSFB) are known as locations of transport of central CNS stimulants. It is necessary to know the kinetics of drugs and caffeine across these two barriers to investigate the relationships between stimulants and caffeine in their transport. There have been few studies of changes in the absorption of caffeine in particular. Although the kinetics of caffeine in the BBB have been reported in some studies [12,13,14], no study has examined the kinetics of caffeine in the BCSFB in detail. It is known that caffeine after transfer through the BCSFB reaches the brain easily [15,16,17], but the details of the kinetics of caffeine on passage through the BCSFB are unknown.

Therefore, to investigate the kinetics of caffeine in the BCSFB and the relationship between caffeine and other CNS stimulants, we developed a BCSFB model using cultured cells and conducted morphological and pharmacological investigations in autopsy cases.

## 2. Results

### 2.1. Number of Cases in Which Stimulants and Psychotropic Drugs Were Detected

Of the 127 cases, stimulants were detected in 30 cases (Group A), and in the remaining 97 cases, stimulants were not detected (Group B).

Furthermore, of the 30 cases in Group A, only stimulants were detected in 17 cases (Group I), whereas both stimulants and psychotropic drugs were detected in 13 cases (Group II). Of the 97 cases in Group B, only psychotropic drugs were detected in 26 cases (Group III). Neither stimulants nor psychotropic drugs were detected in the remaining 71 cases (Group IV).

### 2.2. Caffeine Concentration in Right Heart Blood and CSF

#### 2.2.1. Caffeine Concentration in Groups A and B

**Figure 1:**
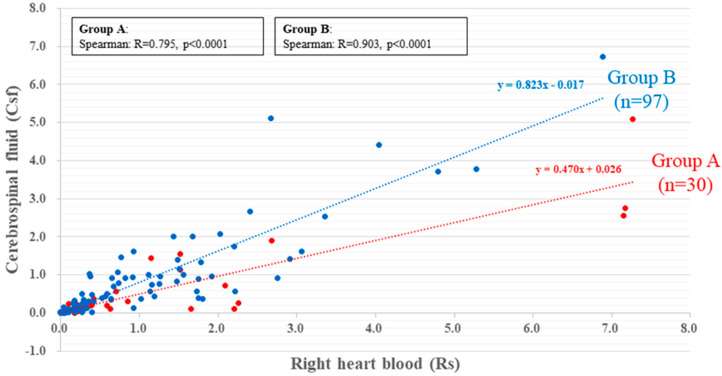
The correlation between the concentration of caffeine in Rs and that in CSF in Groups A (red) and B (blue). There is a correlation between the caffeine concentrations in Rs and in CSF in both groups (Group A: r = 0.795, *p* < 0.0001; Group B: r = 0.903, *p* < 0.0001). The concentration of caffeine is higher in Rs than in CSF in Group A.

Both Groups A and B showed a high correlation between right heart blood (Rs) (x) and CSF (y) caffeine concentrations (Group A: y = 0.470x + 0.026, r = 0.795, *p* < 0.0001; Group B: y = 0.823x − 0.017, r = 0.903, *p* < 0.0001). The distribution of caffeine in the scatter plot was more biased toward the Rs side in Group A than in Group B (Figure 1 and Table 1).

#### 2.2.2. Relationship between Caffein Concentration in Rs and CSF in Groups I, II, III, and IV

**Figure 2:**
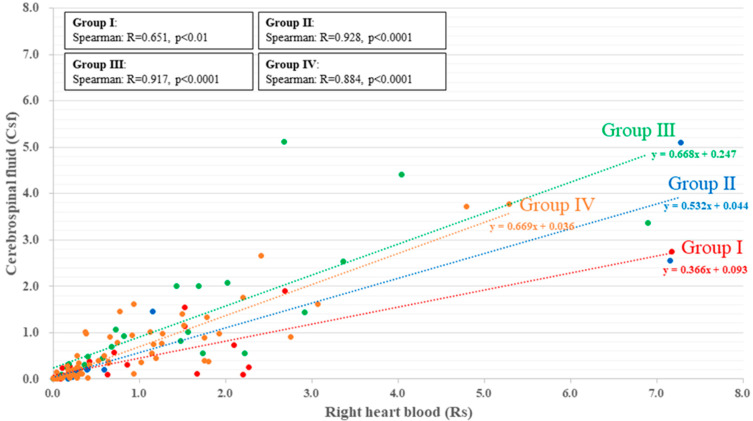
The correlation between the concentration of caffeine in Rs and that in CSF in Groups I (red), II (blue), III (green), and IV (orange). There is a correlation between the concentration of caffeine in Rs and in CSF in all groups (Group I: r = 0.651, *p* < 0.01; Group II: r = 0.928, *p* < 0.0001; Group III: r = 0.917, *p* < 0.0001; Group IV: r = 0.884, *p* < 0.0001). The concentration of caffeine is higher in Rs than in CSF in Groups I and II.

In all four groups, the concentration of caffeine was highly correlated between Rs (x) and CSF (y) (Group I: y = 0.366x + 0.093, r = 0.651, *p* < 0.01; Group II: y = 0.532x + 0.044, r = 0.928, *p* < 0.0001; Group III: y = 0.668x + 0.247, r = 0.917, *p* < 0.0001; Group IV: y = 0.669x + 0.036, r = 0.884, *p* < 0.0001). Caffeine distribution in Groups I and II, in which stimulants were detected, was skewed toward the Rs side compared with Groups III and IV, in which stimulants were undetected. In the comparison between Group I (stimulants) and IV (non-stimulants), caffeine distribution in Group I was greater with the degree of inclination on the Rs side than in Group IV. Moreover, in the comparison between Groups II (stimulants with psychotropics) and III (non-stimulant with psychotropics), caffeine distribution in Group II was greater with the degree of inclination on the Rs side than in Group III (Figure 2 and Table 1).

### 2.3. Permeability of Caffeine and 4-Aminopyridine in the Culture Cell Model of the BCSFB

**Figure 3:**
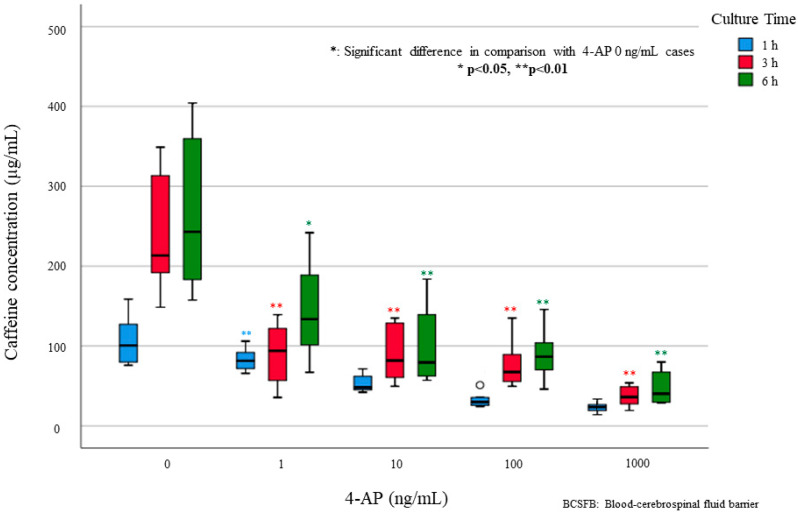
Concentration of caffeine after the addition of 4-AP by the time after administration of each concentration of 4-AP in the BCSFB model.

The experimental results in the culture experimental model simulating the BCSFB are shown in Figure 3. Experiments without 4-AP showed higher caffeine concentration on the CSF side than experiments with high concentrations of 4-AP. In experiments with 4-AP, there was a shift of caffeine concentration to the CSF side over time after administration at any 4-AP concentration focusing on the elapsed time after caffeine and 4-aminopyridine (4-AP) administration. There was a significant shift in the caffeine concentration to the CSF side 1 h and 6 h after administration at any 4-AP concentration. That is, with the increase in the concentration of 4-AP added with caffeine, the concentration of caffeine migrated to the CSF side decreased. In particular, the concentration of caffeine on the CSF side decreased as the concentration of 4-AP added at each elapsed time increased, based on the addition of 0 ng/mL 4-AP (Figure 3 and Table 2).

### 2.4. Morphological Changes in the Choroid Plexus with Drug Administration

**Figure 4:**
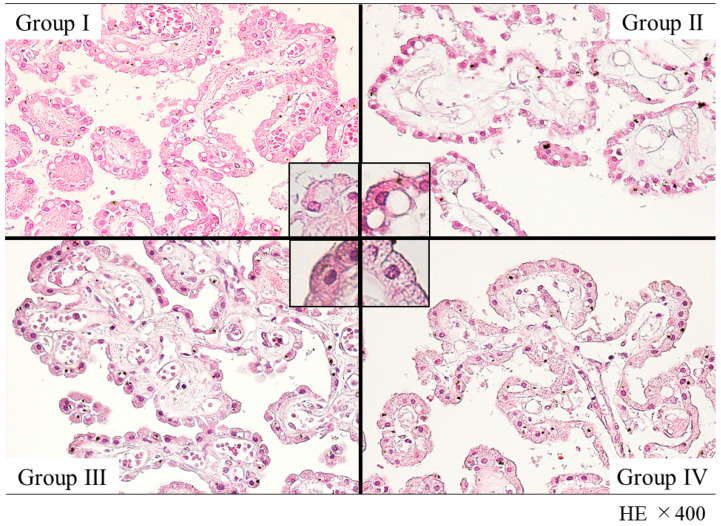
The histopathological findings of choroid plexus epithelial cells in autopsy cases in each group (H&E staining 400×). Cytoplasmic vacuolations are seen. In particular, more than 10% of choroid plexus epithelial cells have cytoplasmic vacuolations in Groups I (27.5%) and II (13.6%), with 9.2% in Group III and 2.1% in Group IV.

**Figure 5:**
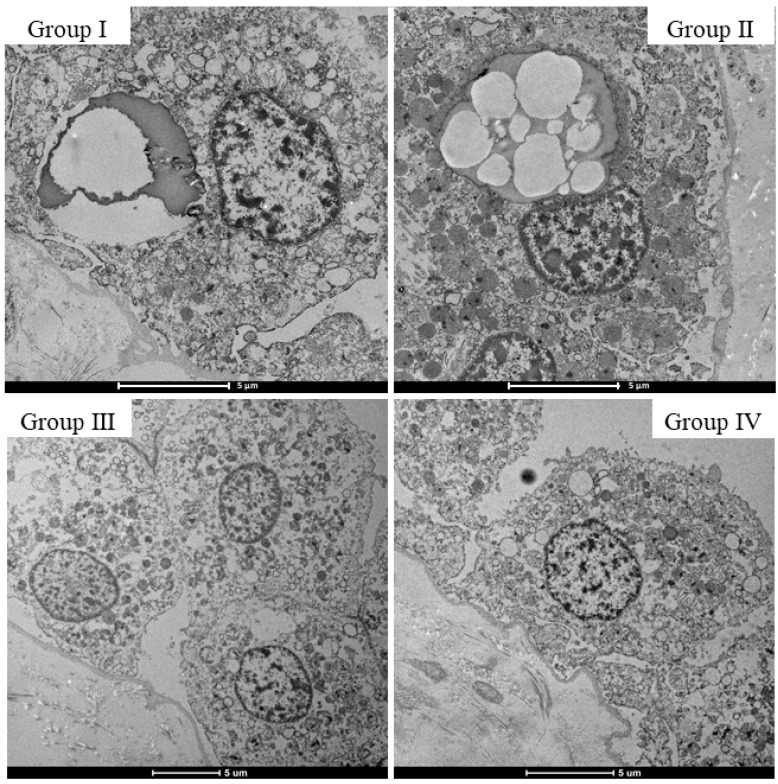
Morphological findings of choroid plexus epithelial cells using TEM. In Groups A and B, cytoplasmic vacuoles of the same size as the nuclei are observed; it seems that something has pooled in the cytoplasmic vacuole.

Hematoxylin and eosin (H&E) staining was performed in the choroid plexus of various groups in which drugs were detected (Groups I–III and Control Group IV), and morphological changes were observed. Multiple vacuoles were observed in choroid plexus epithelial cells, and their number was the highest in Group I (Figure 4). Furthermore, on transmission electron microscopy (TEM) observations of the micromorphology of choroid plexus epithelial cells, vacuoles in Groups I and II were observed, which clearly indicated the presence of material accumulation, whereas in Groups III and IV, almost no vacuoles were observed (Figure 5).

### 2.5. Comparison of Caffeine Concentrations in Choroid Plexus Epithelial Cells and Vascular Endothelial Cells in the BCSFB Model

**Figure 6:**
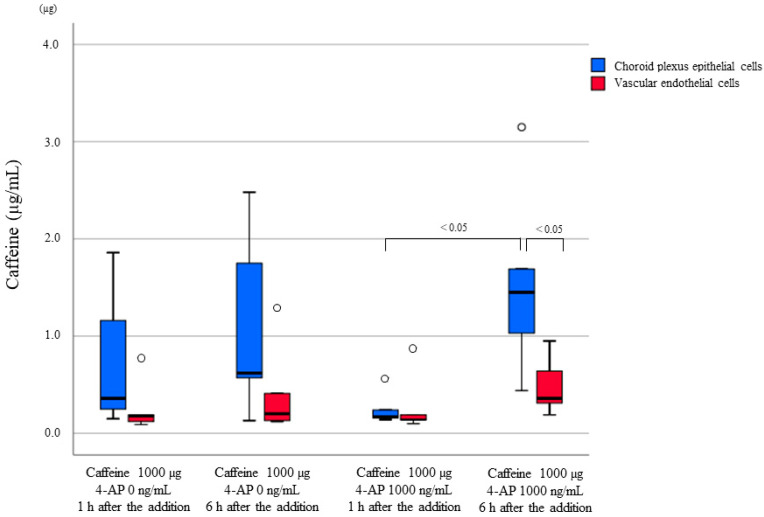
Concentrations of caffeine in vascular endothelial cells and in choroid plexus epithelial cells. Under all conditions, the concentration of caffeine is higher in choroid plexus epithelial cells than in vascular endothelial cells. In particular, a significant difference in caffeine concentration is observed 6 h after 1000 ng/mL 4-AP was added.

To confirm the site of caffeine accumulation, 0 ng/mL and 1000 ng/mL 4-AP were added to the BCSFB model, a cultured cell model, using 1000 µg caffeine as a base, and caffeine concentrations in vascular and choroid plexus epithelial cells of the BCSFB model were measured 1 h and 6 h after addition, respectively. The results showed that choroid plexus epithelial cells had higher levels of caffeine than vascular endothelial cells under all conditions. Notably, a significant difference in the concentration of caffeine was observed between vascular and choroid plexus epithelial cells 6 h after the addition of 1000 ng/mL 4-AP. Moreover, an approximately eight-fold significant difference in the concentration of caffeine was observed in choroid plexus epithelial cells 1 h and 6 h after the addition of 1000 µg caffeine and 1000 ng/mL 4-AP. The difference in the concentration of caffeine between vascular and choroid plexus epithelial cells was the smallest 1 h after the addition of 1000 ng/mL 4-AP (Figure 6 and Table 3).

## 3. Discussion

In this study, CNS stimulants in the BCSFB were found to inhibit the transfer of caffeine from blood to CSF. To clarify the kinetics of caffeine in the BCSFB and the physiological relationships between caffeine and CNS stimulants, this study was conducted using actual autopsy cases for comparison and basic culture experiments. In the examination of autopsy cases, a moderate to high correlation between blood caffeine concentration and CSF caffeine concentration was observed among all drug type groups. When caffeine concentrations in blood and CSF were compared, caffeine was distributed in such a way that its concentration in blood was higher, and this difference was particularly noticeable when stimulants were taken, becoming larger. This suggested that the BCSFB does not allow caffeine to pass through when a CNS stimulant is administered.

Studies have reported that, as a result of comparing caffeine concentrations in blood and CSF, caffeine concentrations were found to be lower in CSF than in blood, although they were correlated with each other [18]. The slope of the correlation between caffeine concentrations in blood and in CSF derived from that study, and the slope of the correlations between caffeine concentrations in blood and in CSF in Group B, Group III, and Group IV (Table 1) in this study, in which no stimulants were detected, were found to be consistent. This is thought to depend on the property that caffeine is absorbed by the brain at a given percentage after intake [18,19]. Considering the results of previous studies, this study suggests that the BCSFB has an unusual function in caffeine uptake when CNS stimulants are taken.

Meanwhile, the function of the BCSFB was investigated by simultaneously adding caffeine and 4-AP, a neuroexcitatory agent, to the BCSFB cultured cell model in vitro. In human autopsy cases, the stimulants are used as neurostimulants; however, in culture experiments, 4-AP is used instead of stimulants. Studies have shown that some cells do not respond to the administration of stimulants into cultured cells unless a large amount of the stimulant is used in the cultured cells, and thus, 4-AP was used [20,21,22]. Although 4-AP is a potassium blocker [23,24], as mentioned earlier, caffeine is a highly lipophilic substance [13] and can easily pass through cell membranes, so we thought that the BCSFB would not be susceptible to the effects of 4-AP. Furthermore, 4-AP increases neurotransmitters [25,26], and since this feature is common to CNS stimulants, 4-AP is used as an alternative to CNS stimulants in culture experiments.

The results of the culture experiments showed that the concentration of caffeine transferred to the CSF side decreased in inverse correlation with the concentration of 4-AP added and increased in proportion to the time elapsed after the addition of 4-AP. Although the concentration of caffeine passing through the BCSFB increases with time, the amount decreases inversely proportionally since the concentration of 4-AP inhibits the transfer of caffeine to the CNS through the BCSFB.

The difference in caffeine concentrations between blood and CSF in the presence of stimulants in the autopsy cases could be due to the inhibition of caffeine transfer to the brain when caffeine passes through the BCSFB. It is the tight junction formed by choroid plexus epithelial cells that is responsible for the barrier function in the BCSFB, which limits the passage of macromolecular substances [15,27]. Moreover, choroid plexus epithelial cells are responsible for secretion, synthesis, reabsorption, metabolism, and uptake of inflammatory cells [27,28,29]. A possible mechanism for inhibiting caffeine transfer in the BCSFB is reabsorption during or after passing through the BCSFB or the enhanced metabolism of some drugs in choroid plexus epithelial cells.

The involvement of transporters is considered when reabsorption increases. In choroid plexus epithelial cells, various transporters responsible for drug transport are present at the apical and basal portions of the choroid plexus [28,30,31]; drug toxins that pass through the cell membrane and are absorbed into CSF are removed; and the nerves are protected by transporters with drug efflux functions [30,31]. Transporters are rich in variety and eliminate anticancer drugs, antimicrobials, and foreign substances in the body from the CNS to the blood side [31]. Although there are no explicit reports of the presence of transporters focused on the elimination of caffeine in choroid plexus epithelial cells, the possibility that caffeine was removed from CSF and reabsorbed into the blood side or choroid plexus epithelium by transporters responsible for caffeine efflux cannot be ruled out.

In contrast, the possibility that caffeine metabolism is enhanced in choroid plexus epithelial cells is considered as follows. Choroid plexus epithelial cells contain nicotinamide adenine dinucleotide phosphate cytochrome P450 reductase, which supplies electrons to CYP enzymes, and drug metabolism by CYP enzymes occurs in choroid plexus epithelial cells, which function as an “enzymatic barrier” [28]. In the CNS, CYP2D6, a group of CYP enzymes, is said to be present in cranial nerves [32,33]; however, whether CYP enzymes are present in choroid plexus epithelial cells is unclear. Moreover, there have been no reports of whether CYP1A2, which is responsible for the metabolism of caffeine [12,13], is present in the CNS of humans; however, its presence has been confirmed in the rat brain [34]. In a study of guinea pigs, methamphetamine increased the activity of CYP1A2 [35]. Based on the assumption that CYP1A2 exists in human choroid plexus epithelial cells, it is possible that methamphetamine also activates CYP1A2 in humans and increases caffeine metabolism, resulting in differences in caffeine concentrations between blood and CSF. However, another study reported that 4-AP, which was used as an alternative to stimulants, neither inhibited nor induced CYP1A2 [36]. In the culture experiments of this study, the inhibition of caffeine transfer to the CSF in the BCSFB was stronger when 4-AP was simultaneously added instead of stimulants, suggesting that the inhibition of caffeine transfer was caused by triggers other than metabolic enzyme activity. These results suggest that the inhibition of caffeine transfer to the CSF is most likely due to reabsorption, not hypermetabolism.

Morphological observation by H&E staining and TEM showed multiple vacuoles formed in choroid plexus epithelial cells, especially in cases in which stimulants were detected, where many relatively large vacuoles were observed. Studies have investigated the specific drug-induced vacuolation in choroid plexus epithelial cells and reported that the toxicity of the drug and the effects of compounds in the drug result in vacuolation [37,38]. However, the vacuoles observed in this study were approximately the same size as the nuclei and showed findings different from those seen in previous reports. Furthermore, in the present study, the culture medium on drug addiction was removed, and the concentrations of caffeine in vascular and choroid plexus epithelial cells were measured. Caffeine concentrations in choroid plexus epithelial cells were higher than in vascular endothelial cells under all conditions. Based on the histological findings and the caffeine concentration results in the cells, it was postulated that the vacuoles observed in this study reflect the accumulation and reabsorption of caffeine into choroid plexus epithelial cells during the process of uptake into the CSF, rather than the toxicity of the drug or the degeneration of choroid plexus epithelial cells caused by the compounds. Caffeine concentration differences between the two cell layers were also smaller when measured 1 h after the addition of 1000 ng/mL 4-AP than those measured at 1 h without the addition of 4-AP, suggesting that multiple doses of the drug delay the capacity of the cells to process the drug.

The inhibition of caffeine absorption in the BCSFB when caffeine and stimulants are administered concurrently appears to be due to the CNS being protected from increased toxicity caused by the interaction of caffeine and stimulants. In terms of the interaction between caffeine and stimulants, animal and culture studies in mice have shown that caffeine enhances stimulant action [9,10,11].

Furthermore, a study has reported that the co-administration of methamphetamine and caffein enhances CNS toxicity more than methamphetamine alone [9]. They are related to the fact that both caffeine and stimulants increase dopamine concentrations [9,39,40,41,42,43] and that dopamine released outside the cell is oxidized, resulting in the formation of reactive oxygen species (ROS) [9,44,45]. Caffeine and stimulants used together may increase the concentration of dopamine and the amount of ROS produced, thereby increasing nerve damage caused by ROS [9]. Caffeine is an adenosine receptor antagonist and a cytoprotective agent [46,47], making it a promising clinical substance; however, it is also a highly toxic and harmful substance when combined with stimulants under certain conditions. According to the findings of this study, caffeine absorption from the blood to the CSF was inhibited when caffeine and stimulants were present simultaneously to minimize nerve damage by regulating the concentration of caffeine in the CSF and brain. It is thought that the BCSFB, particularly choroid plexus epithelial cells, could inhibit and regulate the transfer of substances into the CNS, depending on the substance absorbed. There are two pathways after the passage through the BCSFB: one is through the cerebrospinal fluid–brain barrier, and the other is through the soft membrane to reach the brain after circulation in the subarachnoid space [15,16]; neither of them has a strict barrier function similar to that of the BBB and BCSFB, and the transportation of substances is relatively easy [16,17]. The volume of blood passing through the BCSFB is relatively high, and it is said to be an important structure in the effective exchange of substances between blood and CSF [16]. Despite the large amount of blood flow through the BCSFB, the CSF–brain barrier and pia mater, which exist after the passage through the BCSFB and before reaching the brain parenchyma, cannot be expected to have a strict barrier function, and thus, the barrier function of the BCSFB plays an important role.

In this study, the mechanism of caffeine in the BCSFB and the relationships between drugs and caffeine focusing on the BCSFB were investigated. In the future, we need to investigate the relationships between drugs and caffeine in the BBB, and the differences between caffeine transport through the BBB and that through the BCSFB. These additional investigations will bring us closer to clarification of the mechanisms of drug and caffeine use, and the relationship between drug use and consumption of caffeine.

## 4. Materials and Methods

### 4.1. Autopsy Samples

The cases are shown in Table 4. In this study, 127 autopsy cases (85 males and 42 females), aged 0 to 96 years (median age: 64 years), were examined. Gross findings at dissection, histological findings, and poisoning test results were used to categorize the cause of death. The causes of death were classified into the following 10 types: blunt force injury, sharp instrument injury, asphyxia, drowning, intoxication, fire-related death, heat stroke (hyperthermia), freezing to death (hypothermia), acute cardiac death, and other endogenous deaths. Cases in which there were no complications affecting the study results for each cause of death and the cause of death could be clearly validated were chosen. Blood samples were aseptically collected using a syringe from Rs. CSF was aseptically collected using a syringe from the cisterna at the base of the brain. The collected blood and CSF samples were stored at −80 °C until examination.

### 4.2. Cell Culture Systems and Experiment

#### 4.2.1. BCSFB Model

**Figure 7:**
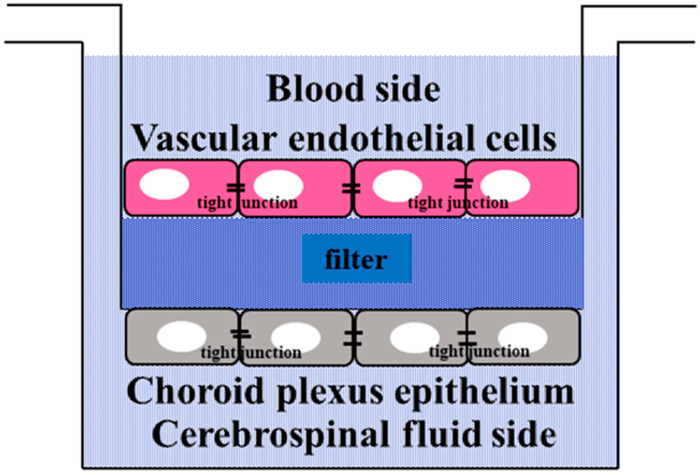
A rough drawing of the BCSFB model.

As BCSFB models, human vascular endothelial cells (ACBR1376) and choroid plexus epithelial cells derived from the human malignant choroid plexus papilloma cell line [48] were used. Both vascular and choroid plexus epithelial cells were cultured with Dulbecco’s Modified Essential Medium/F12 supplemented with 15% inactivated fetal bovine serum, 50 µg/mL streptomycin, 50 µM penicillin, and 0.25 µg/mL fungizone. First, both vascular and choroid plexus epithelial cells were cultured separately at 37 °C, and the cells were cultured on the top and bottom surfaces of the filters, respectively. Initially, choroid plexus epithelial cells were cultured on the bottom surface of the filter. To maintain the monolayers of cells, excessive proliferation on the filter was controlled at 2 × 10^6^/cm^2^. After the formation of tight junctions in choroid plexus epithelial cells, medium exchanges were performed every 2 days. After the choroid plexus epithelial cells were cultured, the top and bottom surfaces of the filter were reversed, and vascular endothelial cells were cultured on the top surface of the filter (opposite choroid plexus epithelial cells), similarly to choroid plexus epithelial cells. Then, the vascular endothelial cell side was dipped up and the choroid plexus epithelial cell side was dipped down into the medium. The containers of the medium were used with an insert (Greiner Bio-One, Frickenhausen, Germany) for 6 well plates with a diameter of 23.1 mm, a pore diameter of 3.0 µm, and a pore density of 2 × 10^6^ holes/cm^2^. Schroten developed this coculture method in a choroid plexus model [49], and it was used in previous studies, including studies on the physiological importance of the BCSFB and prolactin [50,51] (Figure 7).

#### 4.2.2. Culture Experiment

The following experiments were performed using the BCSFB model.

**Figure 8:**
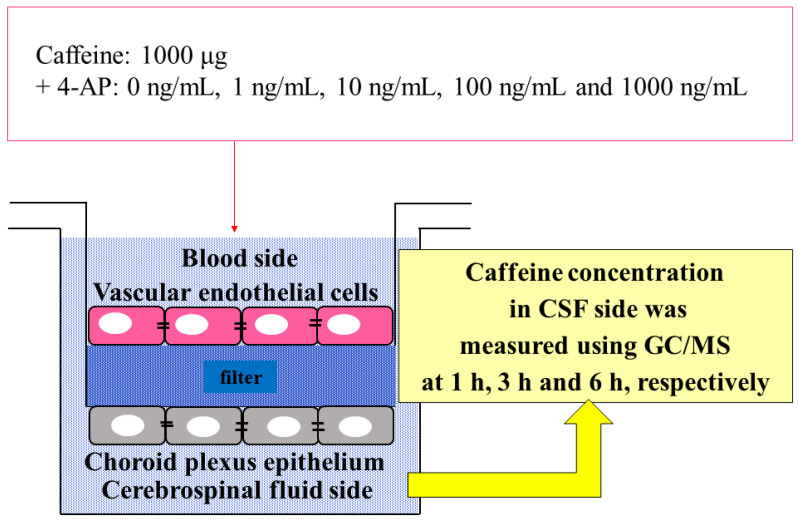
Caffeine and 4-AP administered in the BCSFB model. Caffeine concentration in CSF side was measured using GC/MS at 1, 3 and 6 h, respectively.

In the upper chamber of the BCSFB model (the blood layer on the side of vascular endothelial cells), 1000 µg caffeine and 4-AP, a neuroexcitatory drug, were added at 0, 1, 10, 100, and 1000 ng/mL, and caffeine (caffeine passed through the vascular and choroid plexus epithelial cell layers) concentration in the culture medium accumulated in the lower chamber (the CSF layer on the side of choroid plexus epithelial cells) at 1, 3, and 6 h after each concentration was measured using gas chromatography (GC)/mass spectrometry (MS) (Figure 8). Three samples (two times measurements with GC/MS each sample) were used in this experiment.

#### 4.2.3. Caffeine Concentration in Cultured Cells

**Figure 9:**
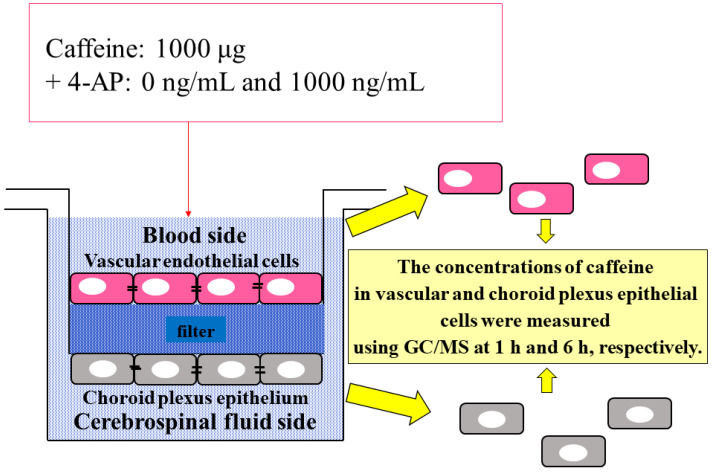
The drug-containing method for measurement under all conditions after the administration of drugs in the BCSFB model. The concentrations of caffeine in vascular and choroid plexus epithelial cells were measured using GC/MS at 1 and 6 h, respectively.

Caffeine concentrations in vascular and choroid plexus epithelial cells were measured 1 and 6 h after the addition of 1000 µg caffeine plus 0 ng/mL and 1000 ng/mL 4-AP, respectively, in the BCSFB model. Each cell was removed from the filter, soaked in 1.3 mL Hanks’ solution, frozen at −80 °C, and thawed at 4 °C. The process was repeated twice. Caffeine concentrations in cells with disrupted cellular structure were measured using GC/MS (Figure 9). Five samples were used in this experiment.

### 4.3. Measurement of Caffeine, Stimulants, and Psychotropic Drugs

#### 4.3.1. Method for Preparing Reagents before Measuring Various Drugs in Autopsy Case Body Fluids and Culture Media

##### Caffeine and Stimulants

The columns used in extracting caffeine and stimulants in the autopsy case body fluids and culture media were Bond Elut Certify (Agilent Technologies, Santa Clara, CA, USA).

A solid powder of caffeine from Sigma-Aldrich (Tokyo, Japan) and methamphetamine hydrochloride from Sumitomo Dainippon Pharma Co. (Osaka, Japan) were used as the standard products. Diazepam-d5 (Sigma-Aldrich, Tokyo, Japan) was used as an internal standard (IS). Deionized pre water was generated using the Milli-Q Purification System (Millipore, Bedford, MA, USA) and was used as distilled water.

As the solvent, 0.1 M phosphate buffer was prepared by dissolving 6.8 g KH2PO4 (Wako, Osaka, Japan) in distilled water at pH 6.0 with potassium hydroxide solution.

Then, 1 M acetic acid was prepared by dissolving 30 mL acetic acid (Wako) in 470 mL distilled water and used as a washing solvent. As one of the eluting solvents, 50 mL dichloromethane (Wako, Osaka, Japan), 20 mL 2-propanol (Wako), and 5 mL ammonia aqueous solution (Wako) were mixed to produce dichloromethane:2-propanol:ammonia (10:4:1).

##### Psychotropic Drugs

The column used in the extraction process during qualitative testing for psychotropic drugs was ISOLUTE^®^ SLE+ 400 µL Sample Vol (Biotage Uppsala, Sweden). During qualitative testing for psychotropic drugs, elution cultures consisting of a 4:1 mixture of dichloromethane and 2-propanol (Wako) were also used.

#### 4.3.2. Extraction Method of Various Drugs

##### Caffeine and Stimulants

Solid-phase extraction was used to extract caffeine and stimulants. First, 500 µL liquid sample was dissolved in 6.0 mL phosphate buffer. Subsequently, for precipitate removal, it was centrifuged at 3000 rpm for 10 min. When the number of tangible components precipitated at the bottom of the container exceeded a given limit, only the supernatant portion, other than the precipitate, was removed and prepared using phosphate buffer after centrifugation. After preparation, 50 µL IS were added at a time, shaken for 10 min, and centrifuged again at 3000 rpm for 10 min. Whether the tangible components did not exceed a given amount was checked again, and the mixture was set on a Gilson GX-274 ASPEC (Middleton, WI, USA) for the extraction process. The samples were loaded onto the column after the inside of the column was pretreated with methanol and 0.1 M phosphate buffer. Subsequently, sequential washing and elution with 1 M acetic acid and dichloromethane were performed. The eluate obtained from this first solid-phase extraction (eluate 1) was evaporated and dried at room temperature under a moderate nitrogen flow. After the first solid-phase extraction, the inside of the column was again pretreated with methanol and 0.1 M phosphate buffer, and then the sample was loaded onto the column, followed by sequential washing and elution with a mixture of 1 M acetic acid and dichloromethane:2-propanol:ammonia (10:4:1), and elution (eluate 2) was performed. Eluate 2 was transferred to a test tube, where the residue from the evaporative drying of eluate 1 was evaporated and dried again at room temperature under a moderate nitrogen flow. To finally obtain the residue of eluates 1 and 2, 50 µL ethyl acetate was added and mixed. The extracts were injected into GC/MS at a volume of 1 µL, and the measurements were performed. Caffeine concentrations were determined by subjecting the blood and CSF samples collected, as well as Hanks’ solution containing culture medium and disrupted cells collected in the culture experiment, to the same extraction processing and caffeine measurement method. For stimulants, the same method was used to measure their concentrations in the blood.

##### Psychotropic Drugs (Psychiatric Drugs)

In this process, 0.25 mL acetonitrile was added to 0.25 mL of the sample and mixed with Precellys^®^ 24 (Bertin Technologies, Montigny-Bretonneux, France). Subsequently, centrifugation at 10,000 rpm was performed for 40 s using a high-speed refrigerated micro-centrifuge (KITMAN, TOMY, Tokyo, Japan) at 4 °C. The column was loaded with 250 µL supernatant solution after centrifugation, followed by 1.3 mL of a 4:1 mixture of dichloromethane and 2-propanol. Then, 1.3 mL dichloromethane was added, and the eluate was evaporated and dried at room temperature with a moderate nitrogen flow. The resulting residue was mixed with 50 µL ethyl acetate, and 1 µL was injected into GC/MS for qualitative testing. Psychotropic drugs identified through qualitative testing were extracted in a manner appropriate for each drug, followed by quantitative testing.

### 4.4. Preparation of Calibration Curves and ISs

Each drug’s working solution for calibration curve preparation was prepared by diluting it with distilled water. The working solution of the IS solution was prepared by diluting 100 µg/mL diazepam-d5 raw solution to a concentration of 10 µg/mL with methanol.

### 4.5. GC/MS Equipment and Conditions

Analysis after solid-phase extraction was performed using Agilent Technologies GC/MS System Model 5975c MSD (column, DB-5MS, 30 m × 0.25 mm i.d.; film thickness, 0.25 mm; column temperature, 100–325 °C (10 °C/min); injector temperature, 280 °C; turbocharged carrier gas, He at a flow rate of 48 cm/s; interface temperature, 300 °C) for quantitative testing.

### 4.6. Histopathological and Micromorphological Changes

After collection, the choroid plexus was fixed in formalin, paraffin-embedded, H&E stained in 4 µm thick sections, and examined under an optical microscope (BX53, Olympus, Tokyo, Japan). H&E-stained specimens had vacuolations in the cells. As a result, under 400× magnification, the percentage of choroid plexus epithelial cells with vacuoles among the entire choroid plexus epithelial cell population was calculated for each drug type.

The following samples were fixed, using TEM to determine whether vacuolation occurs with decay based on the presence or absence of changes in intracellular organelles. The cells were prefixed with 2.5% glutaraldehyde and 2% paraformaldehyde buffered with 0.1 M phosphate buffer (pH 7.4) at 4 °C overnight in a 35 mm Petri dish and then fixed with 1% osmium tetroxide buffered with 0.1 M phosphate buffer for 2.5 h at 4 °C. Ultrathin sections were prepared from resin blocks using a diamond knife on an ultramicrotome (Ultracut UCT, Leica, Vienna, Austria). Sections were stained with 5% uranyl acetate in 50% ethanol for 20 min and Reynolds’ lead citrate for 3 min. Then, the stained sections were analyzed using TEM (Talos F200C G2, Thermo Fisher Scientific, Waltham, MA, USA).

### 4.7. Statistical Analysis

Spearman’s rank correlation coefficient was used to compare two values, including caffeine concentrations in Rs and CSF. The Mann–Whitney U test was used to compare caffeine concentrations in the culture experiment. All analyses were performed using the Statistical Package for the Social Sciences (version 25; IBM Corp., Armonk, NY, USA). *p*-values of less than 0.05 were used to denote significance.

## 5. Conclusions

In this study, it was found that the simultaneous presence of CNS stimulants and caffeine inhibits caffeine transfer to the CSF through the BCSFB, and that choroid plexus epithelial cells plays a key role in this inhibition. The findings of this study will contribute to a better understanding of the kinetics of caffeine in the BCSFB, the addictive mechanism of caffeine consumption, and the mechanism of the correlation between caffeine-containing energy drinks and drugs.

## Figures and Tables

**Table 1:** Relationship between caffeine concentration in blood and that in CSF in each group (*n* = 127).

		Rs Level Range (µg/mL) (Mean/Median)	CSF Level Range (µg/mL) (Mean/Median)	Rs (x) CSF (y)	Rs Level Range (µg/mL) (Mean/Median)	CSF Level Range (µg/mL) (Mean/Median)	Rs (x) CSF (y)
Group A (*n* = 30)	Group Ⅰ: stimulants only (*n* = 17)	0–7.27 (1.40/0.50)	0–5.1 (0.68/0.21)	y = 0.470x + 0.026 (*n* = 30, r = 0.795, *p* < 0.0001)	0–7.17 (1.41/0.85)	0.01–2.75 (0.61/0.27)	y = 0.366x + 0.093 (*n* = 17, r = 0.651, *p* < 0.01)
	Group Ⅱ: stimulants + psychotropic drugs(*n* = 13)				0–7.27 (1.39/0.29)	0–5.1 (0.78/0.18)	y = 0.532x + 0.044 (*n* = 13, r = 0.928, *p* < 0.0001)
Group B (*n* = 97)	Group Ⅲ: psychotropic drugs (*n* = 26)	0–6.89 (0.93/0.4)	0–6.74 (0.75/0.34)	y = 0.823x − 0.017 (*n* = 97, r = 0.903, *p* < 0.0001)	0–6.89 (1.39/0.77)	0–5.12 (1.17/0.63)	y = 0.668x + 0.247 (*n* = 26, r = 0.917, *p* < 0.0001)
	Group Ⅳ: Drug non-detection (*n* = 71)				0–5.28 (0.76/0.31)	0–3.78 (0.55/0.25)	y = 0.669x + 0.036 (*n* = 71. r = 0.884, *p* < 0.0001)

**Table 2:** Changes in the concentration of caffeine (µg/mL) in the CSF side after each 4-AP administration in the BCSFB model.

4-AP Concentration (ng/mL)		0			1			10			100			1000		
Elapsed Time (h)		1	3	6	1	3	6	1	3	6	1	3	6	1	3	6
Sample No.	No. 1	75.82	233.39	404.31	65.81	121.89	241.85	50.35	128.63	139.17	51.04	89.3	98.87	24.17	34.83	67.24
		158.62	313.4	275.88	76.26	100.68	101.4	71.22	49.56	57.08	28.28	48.76	74.4	22.68	27.62	28.88
	No. 2	87.49	191.95	210.16	86.6	87.39	138.38	45.04	98.66	183.76	31.54	55.44	104	33.65	53.83	79.97
		127.16	148.46	157.52	106.1	139.12	188.56	62.02	60.44	75.86	35.58	65.5	46	19	37.08	29.46
	No. 3	113.69	348.85	359.48	71.72	56.69	128.75	42.05	64.81	62.42	25.65	74.21	145.77	26.51	48.98	50.96
		79.8	193.26	183.12	91.86	35.56	66.98	45.84	134.94	82.96	24.06	38.64	70.14	13.94	19.24	29.54

**Table 3:** Caffeine concentrations (µg/mL) in vascular and choroid plexus epithelial cells at different time points after the addition of caffeine and 4-AP.

		Caffeine 1000 µg, 4-AP 0 ng/mL (Control)				Caffeine 1000 µg, 4-AP 1000 ng/mL			
		1 h		6 h		1 h		6 h	
		Vascular Endothelial Cells	Choroid Plexus Epithelial Cells	Vascular Endothelial Cells	Choroid Plexus Epithelial Cells	Vascular Endothelial Cells	Choroid plexus epithelial cells	Vascular Endothelial Cells	Choroid Plexus Epithelial Cells
Sample No.	No. 1	0.77	1.86	1.29	2.48	0.87	0.56	0.36	3.15
	No. 2	0.12	0.25	0.41	1.75	0.19	0.24	0.19	1.69
	No. 3	0.19	0.36	0.2	0.62	0.14	0.16	0.95	1.45
	No. 4	0.18	1.16	0.13	0.57	0.14	0.14	0.64	1.03
	No. 5	0.09	0.15	0.12	0.13	0.10	0.17	0.31	0.44

**Table 4:** Case profiles.

Group		Cause of Death				Sex (M/F)	Age (Mean)	Survival Period		Postmortem Period (Mean, h)
Group A Stimulants detected	Group Ⅰ Stimulants only *n* = 17	Blunt injury	*n* = 1	Fire-related death	*n* = 1	16/1	34–85 (55)	acute	*n* = 10	20.2–61.3 (38)
		Asphyxia	*n* = 3	Hyperthermia	*n* = 1			subacute	*n* = 7	
		Drowning	*n* = 1	Other endogenous death	*n* = 4					
		Intoxication	*n* = 6							
	Group Ⅱ Stimulants + Psychiatric drugs *n* = 13					8/5	26–75 (47.2)	acute	*n* = 4	17.7–61 (38.2)
		Intoxication	*n* = 12					subacute	*n* = 6	
		Blunt injury	*n* = 1					prolonged	*n* = 2	
								unknown	*n* = 1	
Group BStimulants no-detected	Group Ⅲ Psychiatric drugs only *n* = 26	Blunt injury	*n* = 3	Fire-related death	*n* = 4	12/14	3–78 (52.7)	acute	*n* = 7	9.2–63 (36.8)
		Sharp instrument injury	*n* = 3	Other endogenous death	*n* = 4			subacute	*n* = 9	
		Asphyxia	*n* = 3					prolonged	*n* = 8	
		Intoxication	*n* = 9					unknown	*n* = 2	
	Group Ⅳ Drug nondetection *n* = 71	Blunt injury	*n* = 7	Hyperthermia	*n* = 5	49/22	0–96 (62)	acute	*n* = 30	5–64 (33)
		Sharp instrument injury	*n* = 3	Hypothermia	*n* = 4			subacute	*n* = 25	
		Asphyxia	*n* = 13	Acute cardiac death	*n* = 7			prolonged	*n* = 7	
		Fire-related death	*n* = 8	Other endogenous death	*n* = 16			unknown	*n* = 9	

## Data Availability

Data are available upon request from the corresponding author.
